# Human Alphoid^tetO^ Artificial Chromosome as a Gene Therapy Vector for the Developing Hemophilia A Model in Mice

**DOI:** 10.3390/cells9040879

**Published:** 2020-04-03

**Authors:** Sergey V. Ponomartsev, Sergey A. Sinenko, Elena V. Skvortsova, Mikhail A. Liskovykh, Ivan N. Voropaev, Maria M. Savina, Andrey A. Kuzmin, Elena Yu. Kuzmina, Alexandra M. Kondrashkina, Vladimir Larionov, Natalay Kouprina, Alexey N. Tomilin

**Affiliations:** 1 Institute of Cytology, Russian Academy of Sciences, 4 Tikhoretsky Ave., St-Petersburg 194064, Russia; s.ponomartsev@incras.ru (S.V.P.); s.sinenko@incras.ru (S.A.S.); e.skvortsova@incras.ru (E.V.S.); i.voropaev@incras.ru (I.N.V.); nagornaiay-m@mail.ru (M.M.S.); a.kuzmin@incras.ru (A.A.K.); krista-sojka@yandex.ru (E.Y.K.); alex_sandra2502@mail.ru (A.M.K.); 2 Developmental Therapeutics Branch, National Cancer Institute, Bethesda, MD 20892, USA; mikhail.liskovykh@nih.gov (M.A.L.); larionov@mail.nih.gov (V.L.); kouprinn@mail.nih.gov (N.K.); 3 Institute of Translational Biomedicine, St-Petersburg State University, 7–9, Universitetskaya Emb., St-Petersburg 199034, Russia

**Keywords:** human artificial chromosome (HAC), hemophilia, coagulation factor VIII, alphoid^tetO^-HAC, induced pluripotent stem cells (iPSCs), microcell-mediated chromosome transfer (MMCT), cell reprogramming

## Abstract

Human artificial chromosomes (HACs), including the de novo synthesized alphoid^tetO^-HAC, are a powerful tool for introducing genes of interest into eukaryotic cells. HACs are mitotically stable, non-integrative episomal units that have a large transgene insertion capacity and allow efficient and stable transgene expression. Previously, we have shown that the alphoid^tetO^-HAC vector does not interfere with the pluripotent state and provides stable transgene expression in human induced pluripotent cells (iPSCs) and mouse embryonic stem cells (ESCs). In this study, we have elaborated on a mouse model of ex vivo iPSC- and HAC-based treatment of hemophilia A monogenic disease. iPSCs were developed from *FVIII^Y/−^* mutant mice fibroblasts and FVIII cDNA, driven by a ubiquitous promoter, was introduced into the alphoid^tetO^-HAC in hamster CHO cells. Subsequently, the therapeutic alphoid^tetO^-HAC-FVIII was transferred into the *FVIII^Y/–^* iPSCs via the retro-microcell-mediated chromosome transfer method. The therapeutic HAC was maintained as an episomal non-integrative vector in the mouse iPSCs, showing a constitutive FVIII expression. This study is the first step towards treatment development for hemophilia A monogenic disease with the use of a new generation of the synthetic chromosome vector—the alphoid^tetO^-HAC.

## 1. Introduction

A human artificial chromosome (HAC)-based technology developed over the past two decades, represents a technology of engineering, a megabase-sized human artificial vector possessing the main features of a native chromosome, stable episomal maintenance in mammalian cells, high cloning capacity allowing accommodation of the megabase-size genomic loci insertions, and native gene expression [1,2]. In this regard, HACs have become the attractive vectors for gene therapy applications when the provision of a wild-type copy of an affected gene is required. HACs can be built using a top-down approach, i.e., by means of truncation of various human chromosomes [2,3]. Such HACs have been used as high capacity gene therapy vectors in mouse models of human monogenic diseases, including muscular dystrophies [4,5,6,7,8] and hemophilia [9,10] as well as in developing transgenic animals-Cyp-humanized and human antibody-producing mice [11,12,13]. Another type of HAC is synthesized based on the bottom-up approach. This type of HAC is assembled from a synthetic α-satellite DNA array. One of the developed alphoid^tetO^-HAC features is the multiplied tetracycline operator (tet-O) sequence that can bind tet-repressor fusion proteins, leading to a conditional inhibition of the kinetochore function and subsequent HAC loss in dividing cells [14,15,16]. The DNA sequence of the alphoid^tetO^-HAC vector has been shown to exclude any cryptic transcripts [17,18] and demonstrates structural integrity during gene loading and transfer into different host cells [15,19,20,21]. The alphoid^tetO^-HAC features a unique loxP gene acceptor site designed for Cre recombinase-mediated insertion of full-length genes in Chinese hamster ovary (CHO) cells, from which the HAC can be transferred into various recipient cells [22]. All aspects of alphoid^tetO^-HAC applications in synthetic biology, functional genomics, cancer research, and kinetochore investigation have been summarized in the recent review [23]. The alphoid^tetO^-HAC still requires further functional investigation, particularly with respect to the improvement of its mitotic stability. The HAC was also shown to be unstable in some human cell lines and induced pluripotent stem cells (iPSCs) [20,24]. Apart from this, the alphoid^tetO^-HAC has become an attractive gene delivery vector that can be stably maintained in murine embryonic stem cells (ESCs) and their derivatives throughout mouse ontogeny [25] and can also be maintained as a 47th chromosome in human iPSCs [20]. The HAC-based gene therapy approach consists of an ex vivo genetic modification step of the autologous patient-derived stem cells, and consequent infusion of the modified cells into a patient [26,27]. In this study, we have developed a gene therapy model that employs the alphoid^tetO^-HAC as a gene delivery vector in the treatment of the monogenic genetic disease hemophilia A in mice.

Hemophilia A is a monogenic disease caused by a recessive loss-of-function mutation in the human clotting factor VIII (FVIII) gene. Today hemophilia A treatment requires continuous injections with recombinant or plasma-derived FVIII, which has several limitations, including the risk of infection. Gene therapy is an advanced approach for the treatment of this disease, with various strategies previously developed [28,29]. We have developed the alphoid^tetO^-HAC vector bearing the cDNA of the human clotting factor VIII, whose expression can be driven by a constitutive promoter in various mouse tissues. In this work, we have transferred the HAC into iPSCs derived from the *FVIII^Y/–^* mouse and assessed the expression of FVIII in these cells.

## 2. Materials and Methods

### 2.1. Cell Culture

HPRT-deficient CHO cells were cultured in complete DMEM/F12 media: DMEM/F12 (Biolot, Saint-Petersburg, Russia) supplemented with 10% FBS (HyClone, Thermo Fisher Scientific, Waltham, MA, USA), 100 U/mL penicillin, 100 mg/mL streptomycin, 2 mM L-Glutamine (Thermo Fischer Scientific, Waltham, MA, USA). HAC-containing CHO cells were grown in the same medium with the addition of 10 μg/mL blasticidin S (Thermo Fischer Scientific). Mouse iPSCs were cultured in mouse embryonic stem (MES) medium: knock-out DMEM media (Thermo Fisher Scientific, Waltham, MA, USA), 15% FBS (Sigma-Aldrich, St. Louis, MO, USA), 100 U/mL penicillin, 100 mg/mL streptomycin, 2 mM L-Glutamine, 1× non-essential amino acids NEAA (Thermo Fisher Scientific, Waltham, MA, USA), 50 μM beta-mercaptoethanol (Sigma-Aldrich, St. Louis, MO, USA), in-house produced leukemia inhibitor factor LIF (1:5000). Mouse fibroblasts and HEK-293T cells were grown in mouse embryonic fibroblasts (MEF) medium consisting of DMEM media, 10% FBS, 100 U/mL penicillin, 100 mg/mL streptomycin, 2 mM L-Glutamine, and 0.25 μg/mL fungizone (Thermo Fisher Scientific, Waltham, MA, USA).

### 2.2. Vector Construction

To subclone FVIII complementary DNA (transcript variant 1, 7056 bp), the FVIII DNA open reading frame fragment 7080 bp was excised from pUC57-optFVIII (GenScript, Piscataway, New Jersey, NJ, USA) by digesting with XhoI and NheI and cloned into one of the 264-backbone-based plasmids containing the tRNA insulators [19] digested by AvrII and SalI. To replace the CMV sequence to EF1-alpha and cHS4, we excised these elements from plasmid 5760 (details upon request) by digesting with NotI then after 5867 bp DNA fragment treatment with Klenow fragment, it was cut with PacI. The resulting 3106 bp fragment was inserted into 264-backbone-based-optFVIII plasmid by NruI and PacI sites. The resulting plasmid was named EF1-alpha-cHS4-tDNA-optFVIII (details will be provided upon request). Restriction endonucleases and Klenow fragment were from New England Biolab (Ipswich, MA, USA), T4-ligase from Evrogen (Moscow, Russia).

### 2.3. Cre-Recombinase Mediated Insertion of the FVIII Construct into the Alphoid^tetO^-HAC in Hamster CHO Cells

A day before transfection, 3 × 10^4^ of CHO cells with an “empty” HAC (CHO 38.18) were seeded on a 24-well plate with complete DMEM/F12 media. On the day of transfection, the medium was replaced by opti-MEM (Thermo Fisher Scientific, Waltham, MA, USA). Two μg of the FVIII plasmid, 500 ng of the pMC-Cre plasmid, and 5 μL of Lipofectamine 2000 reagent (Thermo Fisher Scientific, Waltham, MA, USA) were mixed by vortexing with 50 μL of opti-MEM, incubated 20 min at RT, and added to the CHO cells. Next, opti-MEM media was replaced by complete DMEM/F12 media after 6 h. After 48 h, the cells were transferred to 10 cm dish with fresh complete DMEM/F12 media supplemented with 10 μg/mL of blasticidin S (Bsd). After 10–12 days of selection, EGFP-positive clones were picked up and plated to a 96-well plate.

### 2.4. Lentivirus Preparations

Lentiviruses in the cell culture supernatant were prepared as previously described [30,31,32,33]. Lentiviruses encoding either the polycistronic cassette containing pluripotency factors Oct4, Sox2, Klf4, cMyc, or rtTA, or EnvΔR were prepared [34,35]. To this end, HEK-293T cells were transfected with envelope-encoding pMD2.G (2.5 μg), packaging psPAX2 (7.5 μg), and either tetO-FUW-OSKM (OSKM), or FUW-M2rtTA (rtTA), or EnvΔR-IRES-TdTomato plasmids (10 μg) by calcium phosphate transfection or polyethylenimine hydrochloride (PEI 40 kDa, 40 μg) transfection method [30]. For the calcium phosphate transfection method, 600 μL of a mixture containing 32.5 μL Tris-EDTA (TE) buffer and 87.5 μL 2M CaCl_2_ with the required amount of DNA was prepared. After vigorous vortexing, 600 mL of HBS buffer (HEPES buffered saline: 280 mM NaCl, 50 mM Hepes, 1.5 mM Na_2_HPO_4_, pH 7.1) was added dropwise. The resulting solution was incubated for 15 min at room temperature (RT) and then added dropwise to the HEK293T cells.

### 2.5. Western Blot

Cells were collected and lysed with 1× SDS gel-loading buffer. Proteins (30 μg of total protein per well) were separated by SDS-PAGE. The proteins were transferred to a nitrocellulose Amersham Hybond-ECL membrane (Thermo Fisher Scientific, Waltham, MA, USA). The membrane was blocked with a solution of 5% dried milk, 0.1% Tween-20 in PBS during 30 min at RT, and incubated with sheep anti-VIII (1:250) (Abcam, Cambridge, MA, USA), mouse anti-actin JLA20-s antibodies (1:500) (DSHB, Iowa City, IA, USA), or rabbit anti-glyceraldehyde 3-phosphate dehydrogenase (GAPDH) (1:1000) (Cell Signaling, Danvers, MA, USA) in 1% milk, 0.1% Tween-20 in PBS overnight at 4 °C. The membranes were washed three times for 5 min with 0.1% Tween-20 in PBS, and incubated with solutions of corresponding peroxidase-conjugated secondary antibodies: donkey anti-sheep, goat anti-rabbit, and goat anti-mouse antibodies (Jackson ImmunoResearch, West Grove, PA, USA), diluted in 1% milk, 0.1% Tween-20 in PBS for 1 h at RT. After washing the membranes three times, they were analyzed using the SuperSignal West Dura Extended Duration Substrate (Thermo Fisher Scientific, Waltham, MA, USA) and Chemidoc Touch Imaging System (Bio-Rad, Hercules, CA, USA).

### 2.6. PCR

The cells were washed with PBS and lysed in 300 μL solution (per well) of 200 μg/mL Proteinase K in 10 mM Tris (pH 7.5), 10 mM EDTA, 10 mM NaCl, 0.5% *N*-lauroylsarcosine for 2 h at 55 °C. The samples were ethanol precipitated by adding 600 μL of ice-cold ethanol, 5% sodium acetate (pH 5.2), and then washed by 70% ethanol. For PCR, we used the HS-Taq PCR-color mix kit (Biolabmix, Novosibirsk, Russia). Five-hundred ng of genome DNA and 10 ng of plasmid DNA per PCR reaction were used. To amplify the EF1-α–FVIII fragment, we used the following primers: forward—5′-acgtcgtctttaggttgggg-3′, reverse—5′-tcggtgaactccacgaacag-3′ (PCR fragment 477 bp). The pUC57-optFVIII plasmid was used as a positive control. To amplify the Cre-recombinase gene, we used the following primers: forward—5′-ccacgaccaagtgacagcaatg-3′, reverse—5′-cagagacggaaatccatccatcgctc-3′ (PCR fragment 373 bp). The pMC-CRE plasmid was used as a positive control.

### 2.7. Preparation of Metaphase Spreads

The metaphase spread was prepared as previously described [20,21,25]. Exponentially growing HAC-carrying CHO or iPSCs were treated for 4 or 12 h at 37 °C with 0.1 μg/mL Colcemid (Sigma-Aldrich) in a 5% CO_2_ incubator. The cells were collected and incubated in hypotonic 0.56% KCl solution for 20 min. Then, the cells were fixed by a solution of methanol/acetic acid (3:1, *v/v*), washed, and stored in the fixative solution at −20 °C. The cell suspension was placed dropwise on the microscope glass slides (Superfrost; Thermo Scientific, Darmstadt, Germany), air-dried, and kept overnight at RT on air.

### 2.8. Fluorescence In Situ Hybridization (FISH) with the PNA Probes

The metaphase spreads slides were incubated with PBS for 15 min at RT, fixed in 4% paraformaldehyde, and thoroughly washed four times with PBS. The slides were consequently dehydrated with 70%, 90%, and 100% ethanol. A hybridization solution containing 10 M Tris–HCl pH 7.4, 70% formamide (Sigma-Aldrich, St. Louis, MO, USA), 5% dextran sulfate, 10 ng tetO PNA-FITC (Panagen Company, Bethel, PA, USA), and 10 ng telomere PNA-TRITS (Panagen Company), was added onto each slide [20]. The slides were covered with coverslips and treated at 80 °C for 3 min and then for 2–6 h at RT in darkness. The slides were consequently washed two times with 70% formamide, 10 mM Tris-HCl (pH 7.4), 0.1% BSA, then three times with 20 mM Tris-HCl (pH 7.4), 136 mM NaCl, 0.08% Tween-20, and rinsed in PBS. The dehydrated slides (as indicated above) were mounted in Vectashield media containing 4’,6-diamidino-2-phenylindole (DAPI) (Santa Cruz Biotechnology, Dallas, TX, USA). The images were captured using the EVOS Cell Imaging Systems (Thermo Fisher Scientific, Waltham, MA, USA).

### 2.9. Southern-Blot Analysis

Southern-blot analysis with the use of a ^32^P-labelled DNA probe was performed as described previously [20,21]. The genomic DNA (5 × 10^5^ cells per sample) was cut by SpeI in an agarose plug. The digested CHEF DNA (CHEF Mapper, Bio-Rad Laboratories, Hercules, CA, USA) was separated on an agarose gel (5–250 kb range, 16 h run), transferred onto a nylon Amersham Hybond-N+ membrane (Thermo Fisher Scientific, Waltham, MA, USA), and hybridized with an alphoid^tetO^-HAC specific 201-bp YAC/BAC DNA probe. The DNA probe was PCR-amplified from the genomic DNA with the use of the ^32^P-labeled dNTPs, and the 5′-GGGCAATTTGTCACAGGG-3′ and 5′-ATCCACTTATCCACGGGGAT-3′ primers. The blot membrane was pre-hybridized at 65 °C for 2 h in Church’s buffer (7% SDS and 0.5 M Na-phosphate buffer) supplemented with 100 μg/mL salmon sperm DNA. The membrane was hybridized with heat-denatured 201-bp YAC/BAC DNA probe at 65 °C overnight. The blot was washed twice with 0.05% SDS, 2xSSC at RT for 10 min, and washed thrice by two times each in 0.05% SDS, 2 × SSC at 60 °C for 5 min, with a reduced concentration of SSC in each subsequent wash (0.5 × SSC, 0.25 × SSC). The blot exposure was at −80 °C for 24–72 h.

### 2.10. Analysis of Mitotic Stability of the FVIII-alphoid^tetO^-HAC in iPSCs

The iPSCs containing the therapeutic HAC were cultivated in media supplemented with blasticidin (4 μg/mL) for 20 days. Then, the cells were grown without blasticidin for another 15 days. At day 0 and 15 after blasticidin withdrawal, the rate of EGFP-positive cells was assessed by flow cytometry, and the rate of HAC loss on the metaphase spreads was counted manually under microscopy 30 metaphase spreads were examined per each cell line. The daily loss rate of the HAC was calculated as previously described [36,37] applying the formula N_15_ = N_0_ × (1 − R)^15^ where N_15_ is the percent of metaphase spreads containing the HAC or EGFP-positive (for FACS assaying) cells after 15 days of blasticidin withdrawal, N_0_ is the percent of metaphase spreads containing the HAC or EGFP-positive cells at day 0 of the blasticidin withdrawal, and R is the daily loss of the HAC.

### 2.11. Murine iPSCs Generation

The primary skin fibroblasts were obtained from the tail tip of *FVIII ^Y/-^* [38], as previously described [25]. In brief, tissue was homogenized with a scalpel and treated with a solution of 1 mg/mL collagenase IV (Sigma-Aldrich, St. Louis, MO, USA) in PBS at 37 °C for 15 min. After washing, the tissue was incubated for 20 min with a 0.05% trypsin/EDTA solution (Thermo Fisher Scientific, Waltham, MA, USA). The cells were cultured in 6-cm plates with MEF medium supplemented with fungizone (Invitrogen) at 37 °С, 5% СО_2_. The cells from 3–4 passages were used for reprogramming. The fibroblasts were seeded in gelatin-coated six-well plates with a cell density of 2 × 10^5^ cells per well. On the next day, 600 μL of the MEF medium and 100 μL of each virus supernatant tetO-FUW-OSKM and FUW-M2rtTA (2.5 × 10^6^ TU/mL) were added. After 6–8 h of incubation, 800 μL of fresh MEF medium was added to the cells. Cells were routinely cultured in mouse embryonic stem cells (MES) medium containing 5 μg/mL doxycycline (Sigma-Aldrich, St. Louis, MO, USA). The medium was replaced every other day; on day 4, the cells were trypsinized and cultured in 10-cm dishes pre-seeded with a mitomycin-inactivated mouse embryonic fibroblast. The iPSC clones were collected and expanded in the MES media without doxycycline.

### 2.12. Teratoma Formation and Histological Analysis

Teratoma analysis was performed as described previously [25,33]. Cells were trypsinized, washed with PBS, and then 10^6^ cells were injected subcutaneously in the hindlimb of NUDE mice. After 3–4 weeks, mice were euthanized, and teratomas were removed and fixed in 4% paraformaldehyde diluted in PBS overnight at 4 °C. Teratomas were cut by pieces with a 5 mm diameter, and dehydrated in a series of ethanol concentrations: 70–80–96%, then with isobutanol and twice in xylene (each step was performed for 1 h at RT). The dehydrated pieces of tissue were incubated once in a solution of 50% paraffin: 50% xylene, and twice in 100% paraffin for 1 h at 56 °C. Paraffin pieces were then cut into 5 μm slices using a microtome Leica RM2235 (Leica Biosystems, Wetzlar, Germany). The paraffin slices were dried at 37 °C overnight on the microscope slides, then washed twice in xylene for 5 min at RT, and rehydrated in a series of ethanol: 96–80–70% for 3 min and in distilled water for 1 min. Next, sections were stained in hematoxylin for 5 min and washed in excess water for 10 min, then stained in eosin for 5 min and washed again in distilled water for 1 min. Sections were dehydrated in a series of ethanol: 70–80–96% for 3 min. Finally, the stained teratoma sections were incubated twice in xylene, mounted with Canadian balsam with a coverslip, and dried at 37 °C overnight. Teratoma histological sections were analyzed using the EVOS Cell Imaging Systems (Thermo Fisher Scientific, Waltham, MA, USA).

### 2.13. Microcell-Mediated Chromosome Transfer (MMCT) Method

MMCT was performed as previously described [20,21,25,35,39] with some modifications. CHO cells carrying the alphoid^tetO^-HAC-FVIII-EGFP were transduced with lentivirus bearing EnvΔR-IRES-TdTomato transgene (MOI = 4, virus titer = 6 × 10^6^ TU/mL). The CHO cells were cultured in T-25 flasks (Greiner, Sigma-Aldrich, St. Louis, MO, USA) covered with 50 μg/mL collagen-I solution (Santa Cruz Biotechnology, Dallas, TX USA) in complete DMEM/F12 media (Biolot, Saint-Petersburg, Russia) supplemented with 10% FBS (Thermo Fisher Scientific, Waltham, MA, USA), 100 U/mL penicillin, 100 mg/mL streptomycin, 2 mM L-Glutamine (Thermo Fisher Scientific, Waltham, MA, USA), and 100 ng/mL colcemid (Wako Pure Chemical, Richmond, VA, USA) at 37 °C for 72 h with a daily media change. CHO cells were treated with DMEM containing 2 μM latrunculin B (Santa Cruz Biotechnology, Dallas, TX, USA) in the T-25 flask for 72 h and centrifuged at 8000× RPM at 34 °C for 1 h using an Avanti HP-26XP, JLA-10.500 rotor (Beckman Coulter Life Sciences, Indianapolis, IN, USA). Microcells were collected and consequently filtered through 8-, 5-, and 3-μm Whatman™ Nuclepore filters (GE Healthcare, Chicago, IL, USA). The microcell fractions were frozen with Cellbanker freezing media (Zenoaq, Tokyo, Japan) at −80 °C [20,39]. Mouse iPSCs were grown in six-well plates up to 70–80% confluence. Cells were trypsinized and collected by centrifugation and gently resuspended in 250 μL warm MES media. Simultaneously, ¼ portion of the prepared microcells containing the alphoid^tetO^-HAC-FVIII-EGFP was defrosted, washed in 10 mL cold DMEM media, and suspended in 50 μL of the warm MES media. Each 250 μL of iPSC suspension and 50 μL of the alphoid^tetO^-HAC-EGFP microcell aliquots were mixed, resuspended carefully, incubated for 10 min at 37 °C, spin, resuspended in 6 mL of MES media, cultivated in a 6-cm dish, and covered with 0.1% gelatin. After 24 h, the medium was replaced with MES media with blasticidin S (3 μg/mL). The selected EGFP-positive clones were grown to 25% confluency, then passed to fresh wells and partially frozen in 0.4 ml of the FreSR™-S media (Stemcell Technologies, Vancouver, BC, Canada).

### 2.14. Fluorescence-Activated Cell Sorting (FACS) Assay of iPSCs

qFACS assay was performed as described previously [20]. The alphoid^tetO^-HAC-FVIII-EGFP iPSCs were grown in MES medium in 6-cm dishes, trypsinized, washed with DMEM/F12, and resuspended in 1 mL of DMEM/F12. Cell sorting of the EGFP-positive and EGFP-negative hiPSCs was done using flow cytofluorimeter EPIX XL (Beckman Coulter, Pasadena, CA, USA). The sorted cells were collected in PBS, centrifuged, and seeded on six-cm plates covered with 0.1% gelatin.

### 2.15. Immunocytochemistry

The cells were grown in 48-well plates, washed with PBS, and fixed in 4% paraformaldehyde in PBS for 10 min [33,40]. The fixed cells were washed with PBS, permeabilized by 0,1% Triton X-100 solution in PBS for 15 min, and incubated with 3% bovine serum albumin (BSA) in PBS for 1 h at RT. The cells were incubated with mouse anti-Oct4 (1:500) (Santa Cruz Biotechnology, Dallas, TX, USA) and rabbit anti-Nanog (1:1000) (Bethyl Laboratories, Montgomery, TX, USA). Antibodies were diluted in PBS solution with 0.1% Tween 20, 3% BSA overnight at 4 °C. After washing the cells with 0.1% Tween 20 in PBS, they were incubated with secondary antibodies in dilution of 1:1000: anti-mouse-Alexa647 and anti-rabbit-Cy3 conjugated (Jackson ImmunoResearch, West Grove, PA, USA) for 1 h at RT. The cells were washed and stained with DAPI in PBS (1:5000), then covered by PBS/sodium azide, and proceeded to an analysis by the EVOS Cell Imaging Systems (Thermo Fisher Scientific, Waltham, MA, USA).

### 2.16. Ethical Statement

All animal procedures were performed according to the guidelines for the humane use of laboratory animals, with standards corresponding to those prescribed by the American Physiological Society. The animal procedures were performed at the Institute of Cytology strictly in agreement with the animal protection legislation acts of the Russian Federation and were approved by the Animal Welfare Assurance of the Institute of Cytology of the Russian Academy of Sciences, Saint Petersburg, Russia, received by Office of Laboratory Animal Welfare, NIH/OD/OER, Bethesda, Maryland, USA, the Assurance Identification number—F18-00380 (period of validity 12.10.2017–31.10.2022).

## 3. Results and Discussion

### 3.1. FVIII Targeting Vector Design

**Figure 1:**
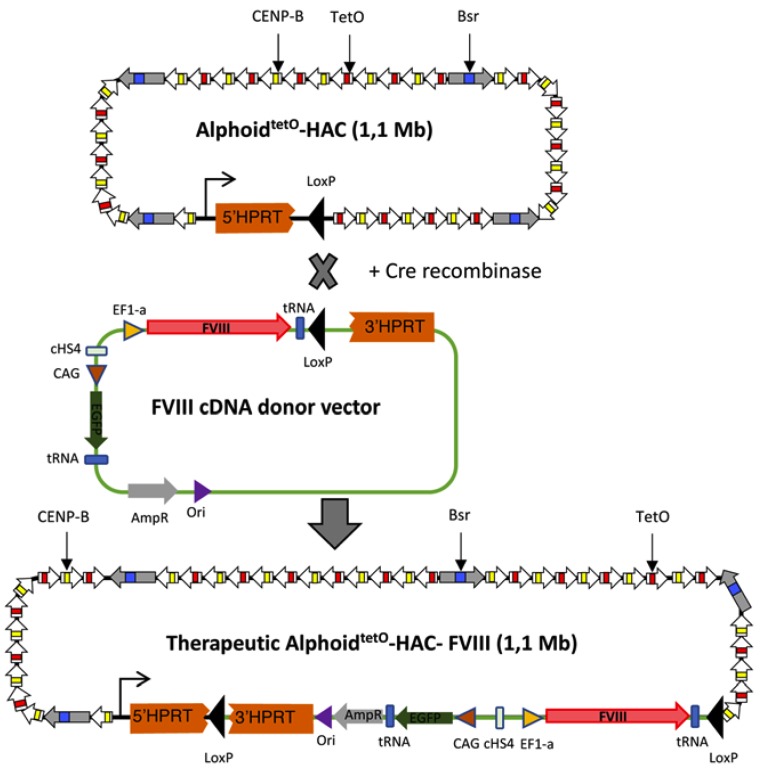
Scheme of construction of the alphoid^tetO^-HAC-factor VIII vector by Cre-recombinase mediated insertion of the therapeutic human clotting factor VIII DNA. After successful Cre-loxP-mediated recombination of the donor vector with the HAC, the *HPRT* gene was reconstructed, allowing positive selection on the HAT medium. tetO—a tet operator site replacing the CENP-B box sequence. Bsr—the blasticidin resistance gene. White arrows with yellow stripe—alphoid monomers with the CENP-B box sequence. White arrows with a red stripe—alphoid monomers with tetO replacing the CENP-B box sequence. 5′HPRT—5′ and 3′HPRT—3′, according parts of the *HPRT* gene. The loxP sites are indicated by black arrowheads. The tRNA insulator indicated by a blue rectangle. cHS4-the insulator indicated by a light green rectangle. The FVIII cDNA -indicated by a red arrow. The EF1-alpha promotor (EF1-α)—indicated by a yellow arrowhead. CAG promoter-indicated by a brown arrowhead. The EGFP gene—indicated by a green arrow). The ampicillin resistance element (Amp^R^)-indicated by a grey arrow. The origin of replication (Ori)-indicated by a purple arrowhead.

To introduce the *FVIII* gene into the alphoid^tetO^-HAC, we constructed a vector featuring a loxP site upstream of the 3′ part of the *HPRT* gene for Cre recombinase-mediated integration (Figure 1). Such integration leads to the restoration of the *HPRT* gene and acquisition of HAT resistance in HPRT-deficient hamster CHO cells, allowing clonal selection. The vector contains the *FVIII* gene under the control of the *EF1α* promoter and the *EGFP* gene under the *CAG* promoter. These two genes were inserted in a “head-to-head” orientation and were separated by the cHS4 insulator to minimize interference of the two strong promoters [41]. Besides, the *FVIII* and the *EGFP* genes have been flanked by the tRNA insulators to prevent their silencing by HAC centrochromatin [19] (Figure 1). The origin of replication and an ampicillin resistance element were also needed to maintain the targeting vector in bacterial cells.

### 3.2. Recombinase-Mediated Insertion of the Targeting Vector into Alphoid^tetO^-HAC

**Figure 2:**
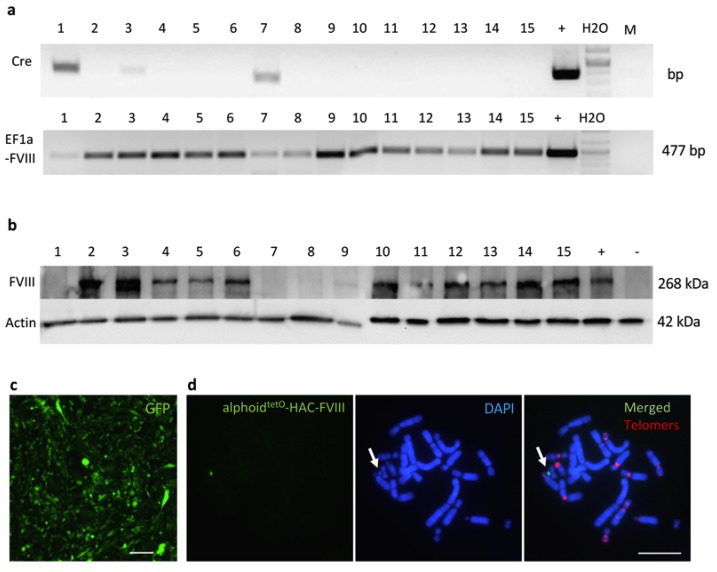
CHO cells with the alphoid^tetO^-HAC-FVIII. (**a**) DNA amplification of the Cre-recombinase gene fragment from the CHO-alphoid^tetO^-HAC-FVIII clones by PCR (upper gel). DNA amplification of the EF1-alpha-FVIII gene fragment from the CHO-alphoid^tetO^-HAC-FVIII cell clones by PCR (lower gel). (+)—the plasmid control. (**b**) Analysis of FVIII protein expression in the CHO-alphoid^tetO^-HAC-FVIII by Western blot. Clones are indicated by numbers. (+)—control CHO cells transfected with the p-FVIII vector. (-)—CHO cells without HAC. (**c**) CHO cells with the alphoid^tetO^-HAC-FVIII expressing EGFP (clone 2) cultured after HAT-selection and BSD-selection, scale bar—100 µm. (**d**) Fluorescence *in situ* hybridization assay of the CHO clone 2 with the alphoid^tetO^ PNA-FITC probe (green) and the telomere PNA-TRITS probe (red). The alphoid^tetO^-HAC-FVIII is indicated by an arrow. Scale bar—10 µm, color-coded markers are indicated on the image.

The targeting vector was loaded into the HAC in HPRT-deficient CHO cells via Cre/lox-recombination (Figure 1). Following the growth in the HAT medium, 15 HPRT-positive CHO clones were selected. PCR analysis revealed that all of them had the *FVIII* gene with the *EF1α* promoter. Twelve clones (Figure 2a) were devoid of the spontaneous Cre-recombinase gene insertions in their genome and, as confirmed by Western blot analysis, expressed the FVIII protein (Figure 2b). Four out of these 12 FVIII-expressing clones were next assessed by FISH analysis, which confirmed that all of them contained an autonomous HAC not integrated into the host chromosomes. One of these clones (clone #2) was used as a HAC donor for further experiments (Figure 2c,d).

### 3.3. iPSC Generation from Fibroblasts of FVIII-Deficient Mice

**Figure 3:**
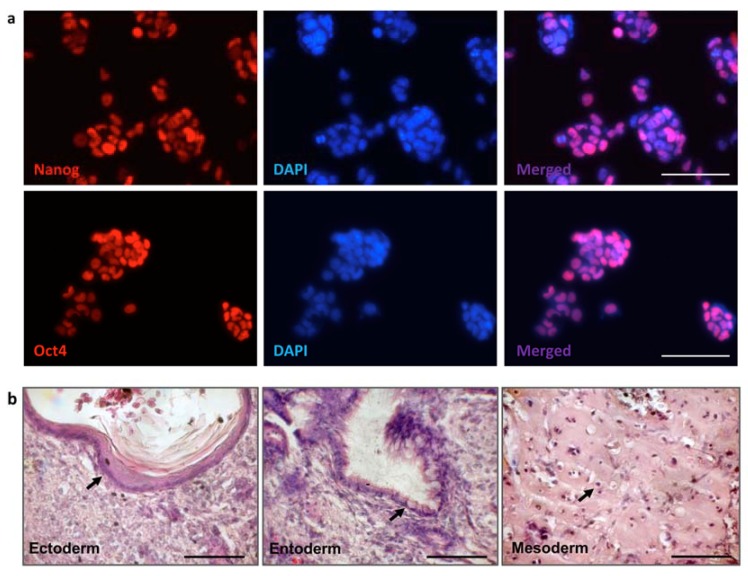
Expression of the pluripotency markers and teratoma formation by induced pluripotent stem cells (iPSCs) developed from *FVIII^Y/-^* fibroblasts. (**a**) Immunostaining of *FVIII^Y/-^* iPSCs with anti-Nanog antibodies (upper panel), and with anti-Oct4 antibodies (lower panel). Color-coded markers are indicated on the panels, scale bar—100 µm. (**b**) Hematoxylin-eosin staining of the *FVIII^Y/-^* iPSC- derived teratomas with developed keratin epidermis of ectoderm, intestine epithelium of entoderm, and cartilage of embryonic mesoderm layers (indicated by arrows), scale bar—100 µm.

Originally, we had selected several recessive genetic disorders for disease modeling: hemophilia A, dysferlinopathy, and X-linked severe combined immunodeficiency (X-SCID), which are characterized by loss-of-function of the FVIII, dysferlin, and a gamma chain of the IL2 receptor, correspondingly. We have derived iPSCs for each of these mutants. However, in this study, we focused entirely on the hemophilia A gene therapy model. For the derivation of murine iPSCs, adult tail-tip fibroblasts from the *FVIII^Y/–^* mice [38] were infected with lentiviruses carrying the doxycycline-inducible polycistronic constructs encoding Oct4, Sox2, Klf4, and c-Myc reprogramming factors and rtTA activator [30,34]. After 16–20 days of reprogramming, 8–10 separate iPSC clones for each mutant were isolated and expanded. The clones were selected based on morphology, growth capacity, and uniform expression of the endogenous Oct4 and Nanog pluripotency markers (Figure 3a). The pluripotency of selected iPSC clones was confirmed based on their ability to develop teratomas containing three embryonic germ layers (Figure 3b). Two independent *FVIII ^Y/-^* iPSC clones were used as HAC recipients.

### 3.4. MMCT of the FVIII-Therapeutic HAC from CHO Cells in Mouse FVIII^Y/–^ iPSCs

To transfer the assembled FVIII-alphoid^tetO^-HAC from CHO cells to iPSCs derived from *FVIII^Y/–^* mouse (clone 2), we used a recently described retro-MMCT method [35] with some modifications [20,25]. The microcells were frozen and kept at −80 °C before fusion with *FVIII^Y/–^* iPSCs. Following the MMCT procedure, *FVIII^Y/–^* alphoid^tetO^-HAC-EF1α-FVIII bearing cells were grown for one week in the medium containing blasticidin (2 μg/mL). Blasticidin was withdrawn, which were then picked up and maintained without blasticidin. At the same time, we also obtained several alphoid^tetO^-HAC-CMV-FVIII-containing *FVIII^Y/–^* iPSC clones by the use of both conventional and retro-MMCT methods. These clones have the episomal HAC vector with FVIII transgene construct driven by the CMV promoter. However, even with early passages, none of these clones showed any FVIII protein expression, as seen by Western blot assay (not shown), indicating that the CMV promoter is not sufficient enough to drive transgene expression in this particular setting. In this regard, we selected only two *FVIII^Y/–^* alphoid^tetO^-HAC-EF1α-FVIII (1 and 2) clones obtained by retro-MMCT for further analysis.

### 3.5. Characterization of iPSC Clones Bearing the Alphoid^tetO^-HAC-EF1α-FVIII

**Figure 4:**
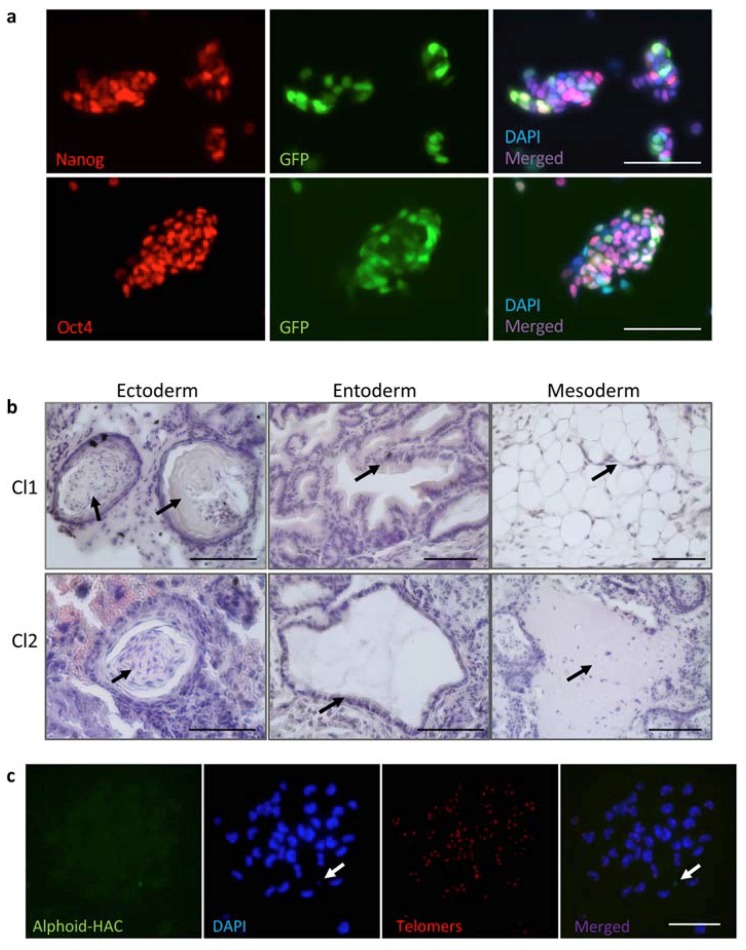
Characterization of *FVIII^Y/-^* iPSCs bearing the alphoid^tetO^-HAC-FVIII. (**a**) Immunostaining of *FVIII^Y/-^* iPSC cells bearing the alphoid^tetO^-HAC-FVIII (clone 2) with anti-Nanog antibodies (upper panel), and with anti-Oct4 antibodies (lower panel). Scale bar—100 µm, color-coded markers are indicated on the panels. (**b**) Clone 1 (Cl1) and clone 2 (Cl2) differentiate in tissues of all three embryonic germ layers in the teratoma assay: keratin epidermis of ectoderm (Cl1 and Cl2); gut epithelium (Cl1) and gland (Cl2) of entoderm; adipocytes (Cl1) and cartilage (Cl2) of mesoderm. Scale bar—100 µm. (**c**) FISH assay of the *FVIII^Y/-^* iPS-HAC-FVIII (clone 2 Cl2) with the alphoid^tetO^ PNA-FITC probe (green) and the telomere PNA-TRITS probe (red). The alphoid^tetO^-HAC-FVIII is indicated by an arrow. Scale bar—10 µm, color-coded markers are indicated on the panels.

**Figure 5:**
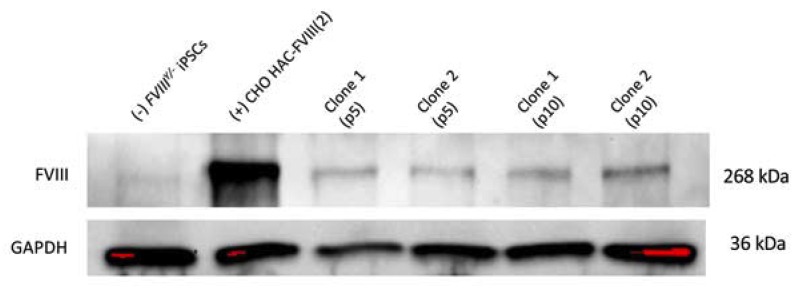
Western blot analysis of FVIII protein expression in the *FVIII^Y/-^* iPSC-HAC-FVIII clone 1 and clone 2 at the 5th (p5) and 10th (p10) passages after cultivation with blasticidin. (–)—a negative control *FVIII^Y/-^* iPSCs without the HAC. (+)—positive control CHO cells bearing the alphoid^tetO -^HAC-FVIII (clone 2).

Two *FVIII^Y/–^ alphoid^tetO^-HAC-EF1α-FVIII* clones obtained by retro-MMCT were positive for EGFP expression and, according to their expression of the pluripotency markers Oct4 and Nanog and ability to form teratomas containing all three embryonic germ layers, retained pluripotent properties (Figure 4a,b). The FISH analysis of both *FVIII^Y/–^ alphoid^tetO^-HAC-FVIII* clones confirmed that the HAC was not integrated into the host genome (Figure 4c) and was maintained as an episomal unit during cell division. We assayed FVIII protein expression in these clones following their cultivation in the standard medium without blasticidin for five and 10 passages. The levels of FVIII protein expression were relatively stable in EGFP-positive cells, selected by flow cytometry during the selected time points (Figure 5). The clones expressed the FVIII protein at a significantly lower level compared to the donor CHO cells. The derived *FVIII^Y/–^* iPSCs cells carrying the alphoid^tetO^-HAC-EF1α-FVIII will be further characterized and proceeded to the differentiation protocols to treat the hemophilia disorder in mice.

**Figure 6:**
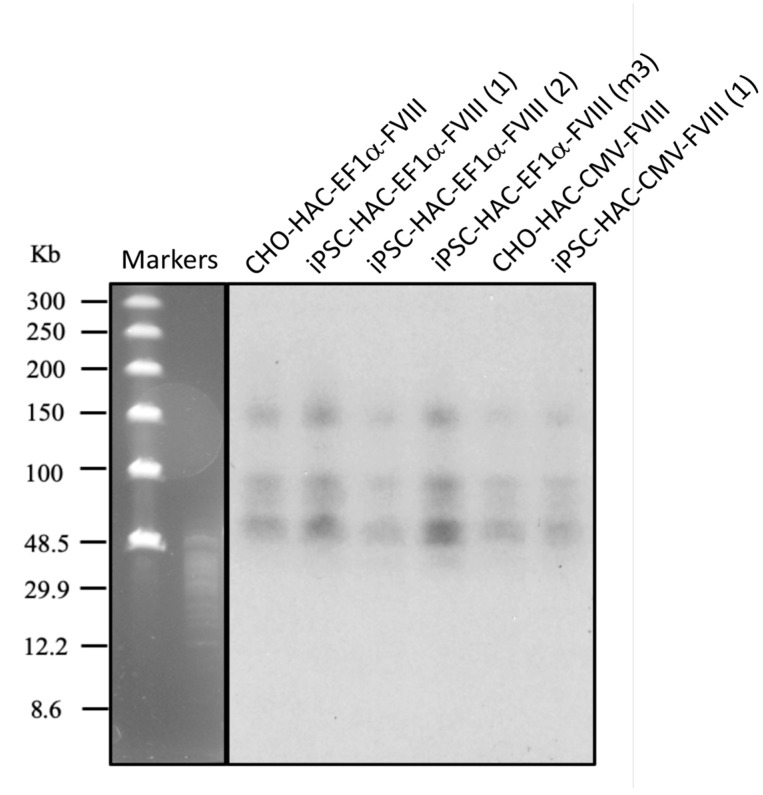
Southern blot assay of the alphoid^tetO^-HAC-FVIII integrity in hamster CHO cells (EF1α-FVIII, CMV-FVIII constructs) and in different *FVIII^Y/-^ iPSC-HAC-*FVIII cell clones: iPSC-HAC-EF1α-FVIII(1), iPSC-HAC-EF1α-FVIII(2), iPSC-HAC-EF1α-FVIII(m3), iPSC-HAC-CMV-FVIII(1). Genomic DNA isolated from alphoid^tetO^-HAC-FVIII containing CHO and iPSCs was digested by SpeI endonuclease cutting the vector part of the HAC and separated by CHEF gel electrophoresis (range 10–300 kb). The blot membrane was hybridized with the ^32^P-labeled tetO-alphoid probe (see Section 2.9). M—Markers: CHEF DNA Size Lambda Ladder (BIO-RAD) and 8–48 kb DNA size standards.

To analyze whether or not the alphoid^tetO^-HAC-FVIII vectors remain structurally intact during the course of MMCT from hamster CHO cells to *FVIII^Y/-^* iPSCs, Southern blot hybridization was performed with the alphoid^tetO^-HAC containing genomic DNA of iPSC clones, digested by SpeI endonuclease (Figure 6). This nuclease cuts the input RCA/SAT43 vector-only once, having no recognition site in the alphoid DNA array of the alphoid^tetO^-HAC [17]. The original alphoid^tetO^-HAC carries 47 copies of the RCA/SAT43 vector utilized for synthetic alphoid DNA array assembly and propagation [16]. The enzyme digested genomic DNA was separated and hybridized with the probe specific to the tetO-alphoid DNA (see Section 2.5 for details). As seen from the Southern blot, a unique pattern of multiple identical bands of different sizes was observed after SpeI cut of the CHO and iPSCs DNAs of different clones, including the construct with CMV promoter (Figure 6). This indicates the absence of any detectable changes in the HAC structure (both for CMV and EF1α promoter constructs) after its MMCT mediated transfer into *FVIII^Y/-^* iPSCs.

**Figure 7:**
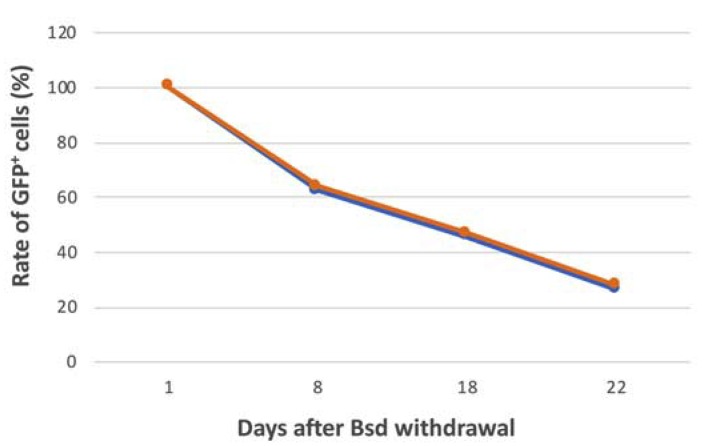
Loss of the HACs in the *FVIII^Y/-^ iPSC-HAC-FVIII* clone 1 (orange line) and clone 2 (blue line) at the 8th, 18th, and 22nd day of culture after Bsd withdrawal, as quantitated by the fluorescence-activated cell sorting assay.

We noticed that after blasticidin withdrawal, some *FVIII^Y/–^ alphoid^tetO^-HAC-EF1α*-*FVIII* iPSCs became EGFP-negative, indicating HAC loss during cell division. This loss was previously observed in human iPSCs bearing the alphoid^tetO^-HAC-EGFP [20]. With this in mind, we compared HAC stability in these two mouse iPSC clones with the previously characterized mouse ESCs bearing the alphoid^tetO^-HAC-EGFP [25] by quantitative FACS and FISH analyses. In addition, we also checked the HAC mitotic stability in the iPSC-HAC-CMV-FVIII clone by FISH analysis. In agreement with the previous results, the alphoid^tetO^-HAC-EGFP was mitotically stable, and in our hands, it showed the daily loss rate—0.008 and 0.004 by FISH and FACS, correspondingly (Table 1 and Table 2), which is consistent with the HAC stability in human HT1080 cells [16]. Interestingly, the daily loss rate of the alphoid^tetO^-HAC-EF1α-FVIII in both *FVIII^Y/–^* iPSCs clones was around 10 times higher (0.04–0.045 and 0.03–0.06, correspondingly) than that of the alphoid^tetO^-HAC-EGFP in ESCs (Figure 7 and Table 1). On the other hand, the mitotic stability of alphoid^tetO^-HAC-CMV-FVIII (daily loss rate—0.006) was comparable to the stability of alphoid^tetO^-HAC-EGFP (Table 2). In this regard, we assume that mitotic stability does not depend on the transgene. Isolation of more alphoid^tetO^-HAC-FVIII clones may allow the identification of the clone with the higher mitotic stability that would be suitable for gene therapy application. In addition, it is known that the mitotic stability of the HACs varies in different types of host cells [24,27,42]. We also cannot exclude that the lower mitotic stability can be associated with the method of HAC transfer because similar poor mitotic stability has been observed for HAC-EGFP human iPSC clones derived with the use of retro-MMCT method [20]. The variability in the mitotic stability of circular HACs [16,24,42] may be due to their ability to form dicentrics by sister HACs recombination. The dicentric HACs are very unstable. At present, we develop an isogenic lineal alphoid-HAC vector, that is likely more stable than the circular HAC due to the presence of the telomere sequences. If a linear HAC is stable in iPSCs, we will use it as a gene therapy vector in our future experiments. Further studies should clarify the cause of the low mitotic stability of the alphoid^tetO^-HAC in certain cells in order to better utilize the HAC for therapeutic applications.

The HAC-based therapeutic model for treatment of hemophilia A was developed by Oshimura’s group [9,10]. A top-down engineered 21HAC2 vector [43] carrying the CAG promoter-driving FVIII cDNA was constructed and maintained in hamster CHO cells and human MSCs. This HAC showed strong and stable *FVIII* transgene expression [9]. In another study, it was shown that *FVIII^Y/–^* iPSC-derived megakaryocytes/platelets carrying the megakaryocyte-specific platelet factor-4 (PF4) promoter-driven FVIII cDNA within the PF4-FVIII-HAC showed a sufficient level of FVIII secretion into the culture medium [10]. Multiple studies demonstrated that de novo synthetized HACs with different transgenes could be delivered to and functionally expressed in various human and murine cell lines [44,45,46,47], as well as successfully used for generation of transgenic mice [48,49,50,51]. Ito and colleagues reported successful treatment of nonalbumine rats by transplantation of immortalized hepatocytes using de novo HAC with the SV40T antigen [50,51]. The alphoid^tetO^-HAC featured a conditional centromere, which can be inactivated by the expression of tet-repressor (tetR) fusion proteins [16,17]. The structure of alphoid^tetO^-HAC has been completely defined, and this HAC is the best characterized among all types of HACs to date [17]. Besides its extensive implementation in various basic chromosome-related studies, this HAC is also being used in terms of its application in gene therapy modeling [15,18,27]. The alphoid^tetO^-HAC was examined for its capacity to carry two average-sized human genes, von Hippel–Lindau tumor suppressor (VHL) and NBS1 protein, to complement genetic deficiencies of cell lines lacking these genes [15]. Here, we have utilized the alphoid^tetO^-HAC for the development of gene therapy using the murine hemophilia A model. The FVIII cDNA was successfully inserted into the alphoid^tetO^-HAC vector, and then the HAC was transferred into mouse iPSCs derived from *FVIII^Y/–^* mutant fibroblasts. Several features of the newly developed HAC-based therapeutic vector require further investigation to improve its quality towards higher mitotic stability and a stable transgene expression. Our results show, as a proof of principle, usage of the alphoid^tetO^-HAC vector in the HAC-based gene therapy model.

## 4. Conclusions

The alphoid^tetO^-HAC represents a de novo synthesized, structurally defined, high capacity episomal vector for fundamental chromosome research and biomedical applications [14,17,52,53,54]. In this study, we utilized this HAC to develop the *FVIII-*carrying therapeutic vector, then delivered this vector into mouse *FVIII^Y/–^* iPSCs and confirmed the stable expression of the transgene in these cells. Thus, the alphoid^tetO^-HAC is a suitable and powerful vector for its implementation in the human hemophilia A disease model in the mouse. Nevertheless, there are still several limitations of HAC-based vectors for gene therapy, which include a low efficiency of HAC transfer to different recipient cells, HAC mitotic instability, an insufficient level of transgene expression in some cells, low efficiency of HAC formation, the complex repeated DNA structure of the HACs, and challenges in amplification of the HAC vector DNA outside of the eukaryotic cells [18,26,27,55,56]. Further investigation is needed to improve HAC vectors for their biomedical therapeutic application.

## Figures and Tables

**Table 1:** The rates of human artificial chromosome (HAC) vector loss during cell division. HAC vectors daily losses determined based on the fluorescence-activated cell sorting assay.

Blasticidin Treatment	mESC-HAC-GFP	iPSC-HAC-F8(Cl1)	iPSC-HAC-F8(Cl2)
20 days with Bsd	97.24%	92.66%	92.52%
15 days without Bsd	91.50%	48.61%	45.78%
HAC daily loss	0.004	0.042	0.045

**Table 2:** The rates of HAC vector loss during cell division. HAC vectors daily losses determined based on quantification of metaphase spreads of the HAC-bearing cells.

Blasticidin Treatment	mES-HAC-GFP	iPSC-HAC-F8(Cl1)	iPS-HAC-F8(Cl2)	iPS-HAC-F8(CMV)
20 days with Bsd	96.67%	96.67%	96.67%	80.00%
At 15 days without Bsd	85.71%	36.73%	59.37%	73.33%
HAC daily loss	0.008	0.062	0.032	0.006
